# Association between HbA1c status relative to individualized targets and estimated 28-day diabetes-related cost in older outpatients with diabetes: a single-center cross-sectional study

**DOI:** 10.1007/s13340-026-00922-5

**Published:** 2026-08-10

**Authors:** Takuya Omura, Takahiro Kamihara

**Affiliations:** 1 Department of Metabolic Research, Research Institute, National Center for Geriatrics and Gerontology, 7-430 Morioka-cho, Obu, Aichi 474-8511 Japan; 2 Department of Diabetes and Endocrinology, Hospital, National Center for Geriatrics and Gerontology, Obu, Aichi Japan; 3 Department of Cardiology, Hospital, National Center for Geriatrics and Gerontology, Obu, Aichi Japan

**Keywords:** Older adult diabetes, Individualized HbA1c target, Hypoglycemia-risk medications, Diabetes-related cost, Cross-sectional study

## Abstract

**Background and aims:**

In older adults with diabetes, HbA1c below the lower limit of individualized targets may reflect intensive or potentially excessive treatment rather than favorable glycemic control. The association between this status and diabetes-related cost remains unclear. We examined the association between HbA1c status relative to individualized targets and estimated 28-day diabetes-related cost.

**Methods:**

This single-center cross-sectional study included 117 outpatients aged ≥ 65 years. Patients were classified as below the lower limit, within/meeting the individualized target, or above the target. Diabetes-related cost, defined as net drug cost plus diabetes-related care management fees, was the primary outcome; net drug cost was analyzed as a component of this outcome. Medication costs were standardized to 28 days. We performed group comparisons and exploratory multivariable regression using ln-transformed diabetes-related cost. Sensitivity analysis using ln(cost + 1) included zero-cost patients.

**Results:**

Nine patients were below the lower limit, 60 were within/meeting the target, and 48 were above the target. Median diabetes-related cost was highest in the below-lower-limit group, followed by the above-target and within/meeting-target groups (27,075, 10,732, and 3,663 JPY/28 days, respectively; *p* < 0.001). In exploratory regression excluding zero-cost patients, the adjusted cost ratio for below-lower-limit versus within/meeting-target status was 2.03 (95% confidence interval, 1.13–3.62; *p* = 0.018). In sensitivity analysis including zero-cost patients, the estimate was imprecise and no longer statistically significant.

**Conclusion:**

Patients below the lower limit of individualized HbA1c targets had the highest unadjusted estimated 28-day diabetes-related cost, with frequent insulin use and care management fees. Medication review may be considered in this subgroup.

**Graphical abstract:**

**Figure:**
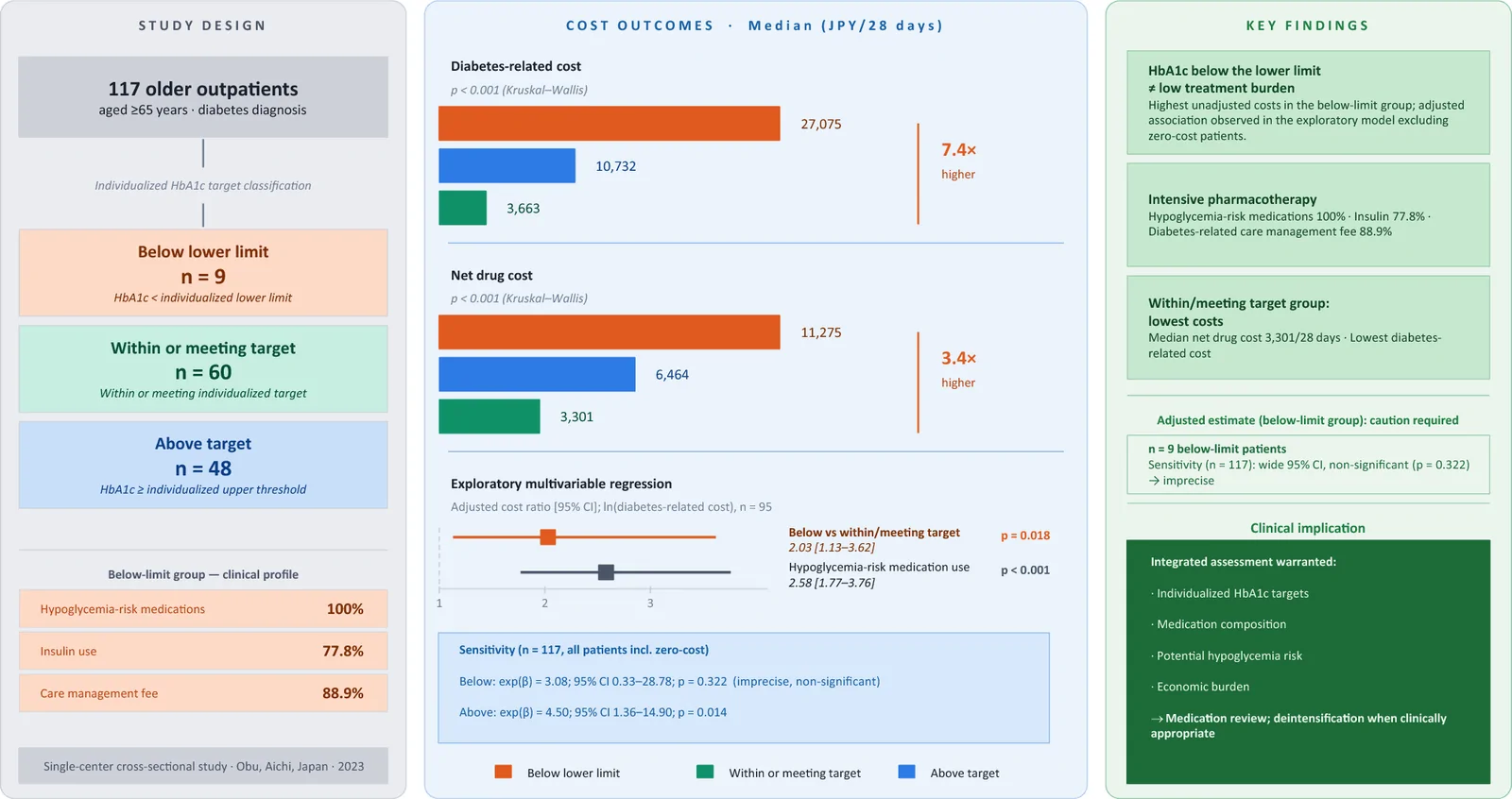

**Supplementary Information:**

The online version contains supplementary material available at https://doi.org/10.1007/s13340-026-00922-5.

## Introduction

Uniform glycemic targets are not appropriate for older adults with diabetes, a heterogeneous population in whom cognitive decline, functional impairment, and polypharmacy complicate treatment decisions [1, 2]. The Japan Diabetes Society and the Japan Geriatrics Society jointly established individualized glycated hemoglobin (HbA1c) targets for older adults with diabetes, stratified by functional category and the presence of medications associated with severe hypoglycemia risk—including insulin, sulfonylureas, and glinides [3].

In this population, both hyperglycemia above the individualized target and HbA1c below a specified lower limit may raise clinical concerns. The lower HbA1c limit in the joint recommendations should be understood not as a uniform treatment target, but as a clinical reference point for individualized decision-making that balances the potential benefits and harms of glycemic treatment. HbA1c below the individualized lower limit may reflect intensive glycemic treatment, particularly when insulin or insulin secretagogues are used. Importantly, intensive or potentially excessive treatment may also be associated with higher treatment costs and management burden, yet this economic dimension has received little attention. With the expanding use of novel agents—including dipeptidyl peptidase-4 inhibitors, sodium–glucose cotransporter 2 inhibitors, and glucagon-like peptide-1 receptor agonists—the relationship between glycemic status, treatment intensity, and cost has become increasingly complex.

Evidence is limited regarding the association between HbA1c status relative to individualized targets and treatment costs in older outpatients with diabetes, particularly when using clearly defined drug-price-based measures that separate medication costs from fixed, non-disease-specific fees. We aimed to examine the association between HbA1c status relative to individualized targets and estimated 28-day diabetes-related cost, while also evaluating net drug cost as a medication-cost component in older outpatients with diabetes.

## Methods

### Study design and participants

This was a single-center cross-sectional study conducted at the National Center for Geriatrics and Gerontology. Geriatric diabetes category classification was performed for outpatients with diabetes whenever possible. As of August 2023, 137 patients had completed geriatric diabetes category classification; after excluding 20 patients aged < 65 years, 117 patients aged ≥ 65 years were included in the present analysis. Clinical and cost information was obtained from the most recent outpatient visit between May 19 and August 22, 2023. Diabetes type was obtained from existing study records and classified as type 1 diabetes, type 2 diabetes, combined type 2 and other specified diabetes, or other specified diabetes. The study was approved by the Institutional Review Board of the National Center for Geriatrics and Gerontology (approval No. 1724; approval date: July 7, 2023). Opt-out consent was used according to institutional policy.

### HbA1c status relative to individualized targets

HbA1c status relative to individualized targets was classified using existing study records based on the joint recommendations for glycemic targets in older adults with diabetes [3]. Patients were categorized according to HbA1c values relative to the target ranges specified in the joint recommendations; this analytic categorization was not intended to judge the appropriateness of treatment in individual patients. Hypoglycemia-risk medications were defined as insulin, sulfonylureas, or glinides.

Patients were categorized into the following three groups: below the lower limit of the individualized HbA1c target, within/meeting the individualized HbA1c target, and above the individualized HbA1c target. For patients using hypoglycemia-risk medications, the lower and upper limits of the individualized HbA1c target range were applied according to the joint recommendations, including age-specific lower limits where applicable. In contrast, for patients not using hypoglycemia-risk medications, no lower HbA1c limit is specified in this framework. Therefore, these patients were classified as meeting the individualized target when their HbA1c was below the applicable upper threshold and as above the individualized target when their HbA1c was at or above that threshold; they were not eligible for classification as below the lower limit.

### Antidiabetic medication use

Antidiabetic medication use at the index visit was assessed from prescription records. Medication classes included insulin, sulfonylureas, glinides, dipeptidyl peptidase-4 inhibitors, oral semaglutide, injectable glucagon-like peptide-1 receptor agonists, sodium–glucose cotransporter 2 inhibitors, metformin/biguanides, imeglimin, thiazolidinediones, and α-glucosidase inhibitors. Medication classes were not mutually exclusive. Incretin-based therapy was defined as dipeptidyl peptidase-4 inhibitor, oral semaglutide, or injectable glucagon-like peptide-1 receptor agonist use.

### Cost definitions

The primary outcome was diabetes-related cost, defined as net drug cost plus diabetes-related care management fees, including home self-injection management fees and blood glucose self-monitoring device-related add-on fees. Net drug cost was analyzed as a component of the primary outcome and was defined as the medication-cost component remaining after fixed consultation and prescription fees were subtracted from the initially calculated 28-day cost estimate. Fixed fees consisted of a consultation fee (JPY 740) and prescription fee (JPY 680). Dispensing fees were not included.

Drug costs were calculated as estimated 28-day costs based on prescription records issued to each patient at the index visit. For non-injectable medications, costs were calculated using the product name, dose, prescribed quantity documented in the prescription, and the corresponding official drug price in the 2023 Japanese National Health Insurance Drug Price Standard (yakka kijun), effective April 1, 2023, as published by the Ministry of Health, Labour and Welfare [4], converted to a 28-day amount where applicable. For injectable preparations, because the dose or amount used per administration varied among patients, costs were estimated using the documented dose or amount used per administration, dosing frequency, the corresponding official drug price in the same drug price standard, and a 28-day period. In this study, all medication costs were standardized to a 28-day period using this single drug price standard.

A supplementary outcome was patient copayment based on net drug cost, calculated by multiplying net drug cost by each patient’s cost-sharing rate. Patients with a cost-sharing rate of 0%, corresponding to public subsidy coverage, were excluded from the copayment analysis.

### Statistical analysis

Continuous variables are presented as medians and interquartile ranges. Categorical variables are presented as numbers and percentages. Group comparisons among the three HbA1c target status groups were performed using the Kruskal–Wallis test for continuous variables. Categorical variables were compared using the chi-squared test when appropriate; Fisher’s exact test, Fisher–Freeman–Halton exact test, or Monte Carlo exact test was used when expected cell counts were small. For post-hoc comparisons of diabetes-related cost and net drug cost, pairwise Mann–Whitney U tests were performed with Bonferroni correction.

For exploratory multivariable modeling, diabetes-related cost was used as the dependent variable because it captures both the medication-cost component and diabetes-related care management fees, thereby reflecting the combined economic burden of pharmacotherapy and disease-management support. Diabetes-related cost was natural-log-transformed for regression analysis. Patients with diabetes-related cost of JPY 0 were excluded because ln transformation was not possible. The independent variables were HbA1c target status, hypoglycemia-risk medication use, geriatric diabetes category, body mass index (BMI), age, and sex. The within/meeting-target group was used as the reference category for HbA1c target status. Because hypoglycemia-risk medication use contributed to the definition of individualized HbA1c targets and is also an important determinant of cost, the adjusted model was interpreted as exploratory.

As a sensitivity analysis, an additional multivariable linear regression model was fitted using ln(diabetes-related cost + 1) as the dependent variable to include patients with diabetes-related cost of JPY 0. Because this model used an ln(cost + 1) transformation, exponentiated coefficients from this sensitivity analysis were not interpreted directly as cost ratios.

Residual normality and multicollinearity were assessed using the Shapiro–Wilk test and the variance inflation factor, respectively. All statistical tests were two-sided, and *p* < 0.05 was considered statistically significant. Analyses were performed using IBM SPSS Statistics, version 29.

## Results

### Patient characteristics

Among the 117 included patients, 9 were below the lower limit of the individualized HbA1c target, 60 were within/meeting the individualized target, and 48 were above the individualized target. Most patients were recorded as having type 2 diabetes. The below-lower-limit group consisted entirely of patients with type 2 diabetes. Patients with type 1 diabetes were present only in the within/meeting-target and above-target groups. The below-lower-limit group was characterized by a high prevalence of hypoglycemia-risk medication use. All patients in this group used at least one hypoglycemia-risk medication, and 77.8% used insulin. Diabetes-related care management fees were also common in this group (Table 1).

**Table 1:** Patient characteristics

Variable	Below lower limit (n = 9)	Within/meeting target (n = 60)	Above target (n = 48)	p value
Age, years	79 [76–81]	79 [73–83]	76 [73–81]	0.543
BMI, kg/m^2^	23.1 [20.9–24.1]	22.3 [20.1–25.3]	22.5 [21.4–25.3]	0.576
HbA1c, %	6.5 [6.3–6.7]	6.7 [6.3–7.6]	8.3 [7.7–8.9]	< 0.001
Male, n (%)	6 (66.7)	38 (63.3)	31 (64.6)	1.000
Diabetes type, n (%)				
Type 1 diabetes	0 (0.0)	1 (1.7)	3 (6.3)	
Type 2 diabetes	9 (100.0)	57 (95.0)	41 (85.4)	
Combined type 2 and other specified diabetes	0 (0.0)	0 (0.0)	2 (4.2)	
Other specified diabetes	0 (0.0)	2 (3.3)	2 (4.2)	
Geriatric diabetes category, n (%)				0.034
Category I	2 (22.2)	34 (56.7)	31 (64.6)	
Category II	4 (44.4)	15 (25.0)	15 (31.3)	
Category III	3 (33.3)	11 (18.3)	2 (4.2)	
Hypoglycemia-risk medications, n (%)	9 (100.0)	24 (40.0)	31 (64.6)	< 0.001
Insulin, n (%)	7 (77.8)	10 (16.7)	17 (35.4)	< 0.001
Sulfonylureas, n (%)	2 (22.2)	15 (25.0)	16 (33.3)	0.660
Glinides, n (%)	0 (0.0)	2 (3.3)	1 (2.1)	1.000
SGLT2 inhibitors, n (%)	4 (44.4)	11 (18.3)	19 (39.6)	0.030
Any diabetes-related care management fee, n (%)	8 (88.9)	13 (21.7)	20 (41.7)	< 0.001

Values are presented as median [interquartile range] or n (%). BMI, body mass index; HbA1c, glycated hemoglobin; SGLT2, sodium–glucose cotransporter 2. P values for continuous variables were calculated using the Kruskal–Wallis test. P values for categorical variables were calculated using the chi-squared test, Fisher’s exact test, Fisher–Freeman–Halton exact test, or Monte Carlo exact test, as appropriate. Diabetes type is presented descriptively and was not subjected to formal statistical testing

The distribution of antidiabetic medication classes is shown in Supplementary Table 1. Incretin-based therapy was used in 6 patients (66.7%) in the below-lower-limit group, 32 patients (53.3%) in the within/meeting-target group, and 30 patients (62.5%) in the above-target group. Injectable GLP-1 receptor agonists were used in 2 patients (22.2%), 4 patients (6.7%), and 5 patients (10.4%), respectively.

In the present dataset, all patients with diabetes-related care management fees used insulin and/or injectable GLP-1 receptor agonists. Among patients with these fees, insulin users accounted for 7 of 8 patients in the below-lower-limit group, 10 of 13 in the within/meeting-target group, and 16 of 20 in the above-target group. The remaining patients with these fees used injectable GLP-1 receptor agonists without insulin.

### Cost outcomes

Diabetes-related cost differed significantly among the three groups. The highest median diabetes-related cost was observed in the below-lower-limit group, followed by the above-target group and the within/meeting-target group. Net drug cost followed the same ordering across groups (Table 2).

**Table 2:** Costs by HbA1c status relative to individualized targets

Outcome	Below lower limit (n = 9)	*Within/meeting* target (n = 60)	Above target (n = 48)	p value
Diabetes-related cost, median [IQR], JPY/28 days	27,075 [15,975–31,332]	3,663 [0–11,582]	10,732 [5,543–20,373]	< 0.001
Net drug cost, median [IQR], JPY/28 days	11,275 [6,993–12,105]	3,301 [0–6,810]	6,464 [3,811–9,842]	< 0.001
Net drug cost, mean ± SD	13,091 ± 14,603	4,439 ± 4,628	7,869 ± 7,173	
Net drug cost, min–max	175–49,405	0–17,898	0–44,032	
Copayment based on net drug cost, median [IQR], JPY/28 days^a^	1,191 [267–1,694]	609 [0–1,357]	1,119 [542–1,876]	0.019

Values are presented as median [interquartile range], mean ± standard deviation, or range. IQR, interquartile range; JPY, Japanese yen; SD, standard deviation. Diabetes-related cost was defined as net drug cost plus diabetes-related care management fees. Copayment based on net drug cost was calculated by multiplying net drug cost by each patient’s cost-sharing rate. ^a^Patients with a cost-sharing rate of 0%, corresponding to public subsidy coverage, were excluded from the copayment analysis; this analysis included 6, 58, and 46 patients in the below-lower-limit, within/meeting-target, and above-target groups, respectively. P values for between-group comparisons of diabetes-related cost, net drug cost, and copayment based on net drug cost were calculated using the Kruskal–Wallis test. Mean ± SD and min–max values are presented as descriptive statistics and were not subjected to formal statistical testing. All costs were standardized to a 28-day period

Bonferroni-corrected pairwise comparisons for net drug cost: below lower limit vs within/meeting target, *p* = 0.025; above target vs within/meeting target, *p* = 0.004; below lower limit vs above target, *p* = 0.636. For diabetes-related cost: below lower limit vs within/meeting target, *p* < 0.001; above target vs within/meeting target, *p* = 0.001; below lower limit vs above target, *p* = 0.037.

### Multivariable regression

In the exploratory multivariable linear regression model using ln(diabetes-related cost) as the dependent variable, 95 patients were included after excluding those with diabetes-related cost of JPY 0. The model showed an R^2^ of 0.415 and adjusted R^2^ of 0.361. Residual normality was acceptable according to the Shapiro–Wilk test (W = 0.983, *p* = 0.271), and multicollinearity was not evident, with a maximum variance inflation factor of 1.58 after excluding the constant term. The adjusted cost ratio relative to the within/meeting-target group was 2.03 (95% confidence interval, 1.13–3.62; *p* = 0.018) for the below-lower-limit group and 1.57 (95% confidence interval, 1.12–2.20; *p* = 0.009) for the above-target group. Hypoglycemia-risk medication use was also strongly associated with higher diabetes-related cost (cost ratio, 2.58; 95% confidence interval, 1.77–3.76; *p* < 0.001) (Table 3).

**Table 3:** Exploratory multivariable linear regression for ln(diabetes-related cost)

Variable	β	Cost ratio [exp(β)]	95% confidence interval	p value
Below lower limit vs within/meeting target	0.706	2.03	1.13–3.62	0.018
Above target vs within/meeting target	0.452	1.57	1.12–2.20	0.009
Hypoglycemia-risk medication use	0.947	2.58	1.77–3.76	< 0.001
Category II vs Category I	0.105	1.11	0.78–1.57	0.552
Category III vs Category I	0.482	1.62	0.94–2.78	0.079
BMI, per 1 kg/m^2^	0.017	1.02	0.97–1.06	0.473
Age, per 1 year	0.004	1.00	0.98–1.03	0.781
Female vs male	− 0.013	0.99	0.69–1.41	0.942

The dependent variable was ln(diabetes-related cost). Patients with diabetes-related cost of JPY 0 were excluded from this model. The within/meeting-target group was used as the reference category for HbA1c target status. Category I and male sex were used as the reference categories for geriatric diabetes category and sex, respectively. BMI, body mass index. Cost ratios and their 95% confidence intervals were calculated by exponentiating regression coefficients and their confidence intervals

### Sensitivity analysis

In the sensitivity analysis using ln(diabetes-related cost + 1) as the dependent variable, all 117 patients were included. In this model, the above-target group was associated with higher transformed diabetes-related cost compared with the within/meeting-target group (exp(β) = 4.50; 95% confidence interval, 1.36–14.90; *p* = 0.014). The estimate for the below-lower-limit group was large but imprecise and was not statistically significant on the transformed-cost scale (exp(β) = 3.08; 95% confidence interval, 0.33–28.78; *p* = 0.322). Hypoglycemia-risk medication use was also associated with higher transformed cost (exp(β) = 44.10; 95% confidence interval, 12.06–161.16; *p* < 0.001). Because this model used an ln(cost + 1) transformation to include patients with zero cost, the exponentiated coefficients should be viewed only as model-based estimates on the transformed-cost scale and not be interpreted as direct cost ratios.

## Discussion

The central finding of this study is that, among older outpatients with diabetes, HbA1c below the lower limit of the individualized target was associated with the highest—not the lowest—estimated 28-day diabetes-related cost in unadjusted comparisons. Median diabetes-related cost in the below-lower-limit group was approximately 7.4-fold higher than in the within/meeting-target group (JPY 27,075 vs JPY 3,663), and median net drug cost was approximately 3.4-fold higher (JPY 11,275 vs JPY 3,301). This pattern persisted for diabetes-related cost in an exploratory multivariable model excluding patients with diabetes-related cost of JPY 0, with a cost ratio of 2.03 for below-lower-limit status versus within/meeting-target status. However, the corresponding estimate was imprecise and not statistically significant in the sensitivity analysis including zero-cost patients, underscoring the need for cautious interpretation. These findings suggest that HbA1c below the individualized lower limit should not be interpreted as a marker of low treatment burden; in this cohort, it identified a subgroup receiving intensive—and costly—pharmacotherapy and related management support.

Because patients with type 1 diabetes generally require insulin therapy, diabetes type is an important consideration when interpreting treatment cost. In the present cohort, however, the below-lower-limit group consisted entirely of patients with type 2 diabetes, and patients with type 1 diabetes were present only in the within/meeting-target and above-target groups. Therefore, the high diabetes-related cost observed in the below-lower-limit group was not explained by an overrepresentation of type 1 diabetes in that group.

The below-lower-limit group, while small (n = 9), was clinically distinctive. All patients used hypoglycemia-risk medications, and 77.8% used insulin. Diabetes-related care management fees were frequent in this group and were observed among patients using insulin and/or injectable GLP-1 receptor agonists. These structural features help explain the disproportionately high diabetes-related cost in this group. From a clinical standpoint, older patients with HbA1c below their individualized lower limit and concurrent use of insulin or insulin secretagogues may warrant careful medication review; treatment deintensification, when clinically appropriate, should be considered in light of potential hypoglycemia risk, treatment burden, and economic cost [5, 6].

The lower HbA1c limit in the joint recommendations should be interpreted as a clinical reference point for individualized decision-making rather than as a uniform treatment target. Thus, HbA1c below this lower limit should not automatically be regarded as inappropriate treatment, but may serve as a signal for medication review in the context of treatment burden, functional status, comorbidities, and economic cost.

The within/meeting-target group had the lowest median net drug cost and diabetes-related cost. This suggests that many patients in the within/meeting-target group were managed with relatively low medication burden and without high-cost diabetes-related management support. However, because this was a cross-sectional study, these findings should not be interpreted as evidence that lower treatment cost leads to target achievement.

The above-target group had intermediate costs, which were significantly higher than those in the within/meeting-target group. This group may include patients with persistent hyperglycemia despite treatment intensification. The relatively high use of sodium–glucose cotransporter 2 (SGLT2) inhibitors and insulin in this group may reflect attempts to improve glycemic control, although causality cannot be inferred.

The multivariable analysis showed that both below-lower-limit status and above-target status were associated with higher diabetes-related cost compared with within/meeting-target status. Hypoglycemia-risk medication use was also strongly associated with higher diabetes-related cost. This association should be interpreted carefully. Sulfonylureas and glinides are relatively inexpensive medications; therefore, the observed association is unlikely to indicate that all hypoglycemia-risk medications contributed equally to higher cost. Rather, the cost signal was likely driven primarily by insulin use and related diabetes-management support. In this dataset, diabetes-related care management fees occurred in the context of injectable therapy; most patients with these fees were insulin users, and the remainder used injectable GLP-1 receptor agonists without insulin. Thus, higher diabetes-related cost in the below-lower-limit group likely reflects treatment structure and management intensity, particularly insulin therapy and injectable treatment support, rather than the use of insulin secretagogues themselves.

The sensitivity analysis including zero-cost patients yielded a similar direction of association for the below-lower-limit group, but the confidence interval was wide and the association was no longer statistically significant. This finding indicates that the adjusted estimate for this group was statistically imprecise.

This study has several limitations. First, the cross-sectional design precludes causal inference; in particular, the direction of the relationship between treatment intensity and HbA1c target status cannot be established. Second, the below-lower-limit group included only 9 patients, substantially limiting statistical precision and power for subgroup analyses. Third, costs were estimated from drug price standards and did not include pharmacy dispensing fees; they therefore do not represent actual out-of-pocket expenditure or total medical costs. Fourth, HbA1c target classification relied on existing study records without independent validation against claims data. Fifth, important confounders—including cognitive status, activities of daily living, history of hypoglycemia, and frailty—were not fully adjusted for and may have influenced the observed associations. In addition, data on hypoglycemia, falls, and hip fractures were not systematically collected. Therefore, we could not determine whether patients below the lower limit of their individualized HbA1c target actually experienced higher rates of these adverse clinical events. The analytic dataset also did not include structured information on hypertension, dyslipidemia, diabetes duration, renal function, diabetic complications, or cardiovascular disease. These factors may influence treatment goals, medication selection, and clinical interpretation in older adults with diabetes; therefore, residual confounding by comorbidities and complications remains possible. Sixth, because hypoglycemia-risk medication use contributed to the definition of individualized HbA1c targets and was also closely related to cost, the adjusted regression analysis should be interpreted as exploratory. Seventh, the primary regression analysis excluded patients with diabetes-related cost of JPY 0 because ln transformation was not possible; although a sensitivity analysis using ln(cost + 1) included all patients, the exponentiated coefficients from this model cannot be interpreted directly as cost ratios. Eighth, because the study was restricted to patients who had undergone geriatric diabetes category classification at a single specialist center, selection bias is a concern: patients who were referred for or completed this structured assessment may have been more functionally impaired, had more complex treatment regimens, or been subject to more intensive clinical management than the broader population of older outpatients with diabetes. Additionally, the National Center for Geriatrics and Gerontology is a tertiary referral institution, and the case mix may differ substantially from that of community-based clinics or primary care settings. Consequently, the observed cost distributions and associations may not be generalizable to all older outpatients with diabetes managed outside specialist geriatric care.

## Conclusion

In older outpatients with diabetes, those below the lower limit of their individualized HbA1c target had substantially higher estimated 28-day diabetes-related cost and net drug cost than those in the within/meeting-target group in unadjusted analyses. In an exploratory regression model excluding patients with diabetes-related cost of JPY 0, below-lower-limit status was associated with higher diabetes-related cost, but the sensitivity analysis including zero-cost patients showed that this adjusted estimate was imprecise and no longer statistically significant. These higher unadjusted costs were accompanied by frequent insulin use, other hypoglycemia-risk medication use, and diabetes-related care management fees. Because adverse clinical events were not assessed, HbA1c below the lower limit should be interpreted as a potential signal for comprehensive medication review rather than as evidence of excess hypoglycemia-related events. These findings support integrated assessment of individualized targets, medication composition, treatment burden, functional status, comorbidities, and economic cost in older adults with diabetes.

## Supplementary Information

Below is the link to the electronic supplementary material.

Supplementary Table 1
