# Supplementary material for: Association between HbA1c status relative to individualized targets and estimated 28-day diabetes-related cost in older outpatients with diabetes: a single-center cross-sectional study
Source: Diabetol Int. 2026 Aug 10;17(4):67. doi: 10.1007/s13340-026-00922-5 (PMC13457750; doi:10.1007/s13340-026-00922-5)
Supplement: Supplementary file 1 [file 13340_2026_922_MOESM1_ESM.docx]

**Supplementary Table 1. Antidiabetic medication use by HbA1c status relative to individualized targets**

| **Medication class** | **Below lower limit (n=9)** | **Within/meeting target (n=60)** | **Above target (n=48)** |
| --- | --- | --- | --- |
| Insulin | 7 (77.8) | 10 (16.7) | 17 (35.4) |
| Sulfonylureas | 2 (22.2) | 15 (25.0) | 16 (33.3) |
| Glinides | 0 (0.0) | 2 (3.3) | 1 (2.1) |
| DPP-4 inhibitors | 4 (44.4) | 26 (43.3) | 21 (43.8) |
| Oral semaglutide | 0 (0.0) | 2 (3.3) | 4 (8.3) |
| Injectable GLP-1 receptor agonists | 2 (22.2) | 4 (6.7) | 5 (10.4) |
| Any GLP-1 receptor agonist | 2 (22.2) | 6 (10.0) | 9 (18.8) |
| Any incretin-based therapy | 6 (66.7) | 32 (53.3) | 30 (62.5) |
| Metformin/biguanides | 2 (22.2) | 14 (23.3) | 14 (29.2) |
| Imeglimin | 0 (0.0) | 2 (3.3) | 1 (2.1) |
| SGLT2 inhibitors | 4 (44.4) | 11 (18.3) | 19 (39.6) |
| Thiazolidinediones | 0 (0.0) | 4 (6.7) | 3 (6.3) |
| α-glucosidase inhibitors | 1 (11.1) | 5 (8.3) | 7 (14.6) |

Values are presented as n (%). Medication classes are not mutually exclusive. DPP-4, dipeptidyl peptidase-4; GLP-1, glucagon-like peptide-1; SGLT2, sodium–glucose cotransporter 2. Incretin-based therapy was defined as DPP-4 inhibitor, oral semaglutide, or injectable GLP-1 receptor agonist use.
